# A concept for major incident triage: full-scaled simulation feasibility study

**DOI:** 10.1186/1471-227X-10-17

**Published:** 2010-08-11

**Authors:** Marius Rehn, Jan E Andersen, Trond Vigerust, Andreas J Krüger, Hans M Lossius

**Affiliations:** 1 Norwegian Air Ambulance Foundation, Drøbak, Norway; 2 Akershus University Hospital, Lørenskog, Norway; 3 Norwegian Air Ambulance, Drøbak, Norway; 4 Department of Anaesthesia and Emergency Medicine, St. Olav University Hospital, Trondheim, Norway; 5 Department of Surgical Sciences, University of Bergen, Bergen, Norway

## Abstract

**Background:**

Efficient management of major incidents involves triage, treatment and transport. In the absence of a standardised interdisciplinary major incident management approach, the Norwegian Air Ambulance Foundation developed Interdisciplinary Emergency Service Cooperation Course (TAS). The TAS-program was established in 1998 and by 2009, approximately 15 500 emergency service professionals have participated in one of more than 500 no-cost courses. The TAS-triage concept is based on the established triage Sieve and Paediatric Triage Tape models but modified with slap-wrap reflective triage tags and paediatric triage stretchers. We evaluated the feasibility and accuracy of the TAS-triage concept in full-scale simulated major incidents.

**Methods:**

The learners participated in two standardised bus crash simulations: without and with competence of TAS-triage and access to TAS-triage equipment. The instructors calculated triage accuracy and measured time consumption while the learners participated in a self-reported before-after study. Each question was scored on a 7-point Likert scale with points labelled "Did not work" (1) through "Worked excellent" (7).

**Results:**

Among the 93 (85%) participating emergency service professionals, 48% confirmed the existence of a major incident triage system in their service, whereas 27% had access to triage tags. The simulations without TAS-triage resulted in a mean over- and undertriage of 12%. When TAS-Triage was used, no mistriage was found. The average time from "scene secured to all patients triaged" was 22 minutes (range 15-32) without TAS-triage vs. 10 minutes (range 5-21) with TAS-triage. The participants replied to "How did interdisciplinary cooperation of triage work?" with mean 4,9 (95% CI 4,7-5,2) before the course vs. mean 5,8 (95% CI 5,6-6,0) after the course, p < 0,001.

**Conclusions:**

Our modified triage Sieve tool is feasible, time-efficient and accurate in allocating priority during simulated bus accidents and may serve as a candidate for a future national standard for major incident triage.

## Background

A major incident has occurred when incident location, severity, type or number of victims require extraordinary resources. Major incidents are heterogeneous by nature and their unexpectedness favours an "all-hazards" approach. Since rescue capacity varies within systems, a major incident for a rural emergency service may not apply to a larger urban emergency service [1]. Rapid access to advanced major incident management have proven to optimize resource use and improve patient outcome [2].

Major incident management involves responders from multiple rescue services and it traverses geographical and jurisdictional lines. Further, it involves multiple tasks such as leadership, preparation, risk-evaluation, triage, treatment and transport. Structuring and standardising these initiatives seems essential given the multitude of responders.

In the absence of a consistent and interoperable national system for major incident management in Norway, the Norwegian Air Ambulance Foundation developed Interdisciplinary Emergency Service Cooperation Course (TAS), a no-cost training concept for all emergency services throughout the country. Since the TAS-program was initiated in 1998, approximately 15 500 professionals have participated in one of more than 500 courses. The TAS-courses has gradually evolved and the principles for disaster health education as proposed by World Association for Disaster and Emergency Medicine has successively been adapted [3]. Major incidents require systems that allow providers to follow their daily pattern of behaviour: the "doctrine of daily routine". The TAS-concept train local inter-disciplinary cooperation and focus on simple field-friendly techniques.

**Figure 1:**
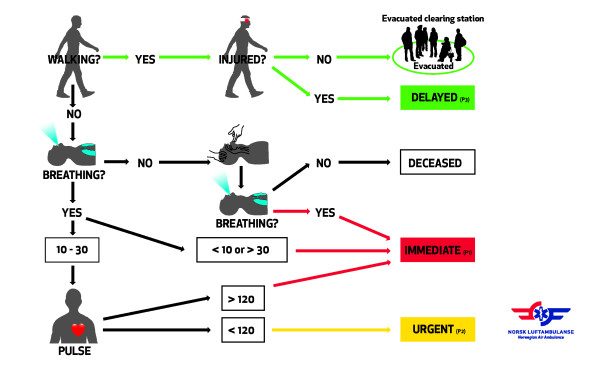
**Modified triage sieve action card**. Adult (>140 cm) triage sieve.

**Figure 2:**
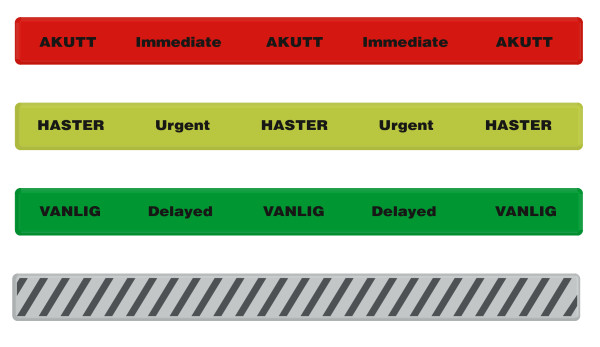
**Reflective slap wrap triage tags**. (P1) immediate (red); (P2) urgent (yellow); (P3) delayed (green) and deceased (white/black).

Acknowledging that triage is necessary to achieve the greatest good for the most number of people [4], we developed a concept for major incident triage based on the established triage Sieve and Paediatric Triage Tape (PTT) models [1,5]. Although several triage tools exists [6,7], the triage Sieve provided an off-the-shelf tool already taught in Major Incident Medical Management and Support (MIMMS) courses in two neighbouring countries (UK and Sweden). The triage Sieve is a major incident primary field triage tool constructed to prioritize patients for evacuation to definitive medical care. Based on the assessments of the ability to walk, airway patency, respiratory- and heart rate, the triage Sieve assigns four priorities: (P1) immediate (red), (P2) urgent (yellow), (P3) delayed (green) and deceased (white/black) [1]. To increase field-friendliness, we designed weather-proof action card (figure 1) and slap wrap reflective triage tags (figure 2).

**Figure 3:**
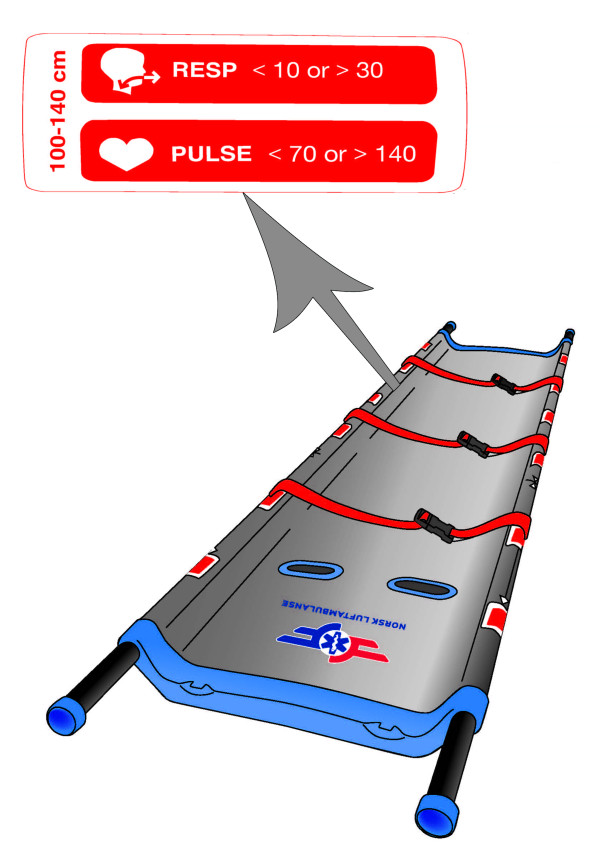
**Paediatric triage tape stretcher**. Details: paediatric vital signs reference values.

The PTT relates a child's supine length to age-related changes in physiological values to overcome the overtriage that occurs when children are subject to the adult triage Sieve algorithm [5]. We designed a tape that presents vital data intervals along the side of stretchers to ensure field-friendly access to the paediatric triage algorithm (figure 3). All children in need of stretchers are allocated (P2) urgent (yellow), but are upgraded to (P1) immediate (red) priority when vital signs lie outside their length-related reference values [8].

The study hypothesis was that learners would improve in speed, triage accuracy and self-efficacy after the TAS-course. We describe the feasibility of a concept for major incident triage and present the accuracy of the modified triage Sieve in full-scaled simulated major incidents.

## Methods

### TAS-course

In the period March-May 2010, TAS-courses were conducted in 4 municipalities with mixed urban/rural and coastal/inland characteristics. Local emergency service personnel (healthcare, police, fire and rescue technicians) were taught major incident self-safety, triage, patient evacuation, extrication techniques and cooperation during a no-cost two-day course. The didactic programme combines theoretical and practical sessions and is tailored to groups of various size and professional composition. A major incident was simulated outdoors using a standardised bus crash scenario including approximately 20 patients (range 17-21) and a real-size bus wreck. Every patient was given an information card (additional file 1) with injury descriptions as well as numeric vital signs for triage purposes. Physiological parameters were dynamic to mimic de-compensation and to visualize the need for re-triage. The patients were equally distributed between the four priorities (all categories had 25% representation). Paediatric patients were simulated with mannequins for ethical reasons. The bus-crash scenario was simulated once at the beginning of the course (no formal triage Sieve competence/no access to TAS-triage equipment) and once at the end of the course (with formal triage Sieve competence/access to TAS-triage action cards, triage tags and paediatric triage stretcher). The didactic program was piloted and refined through 43 TAS-courses prior to the study.

### Study design

**Figure 4:**
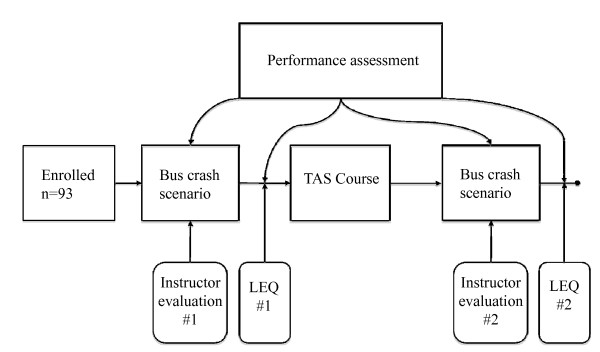
**Study design**. LEQ = Learners Evaluation Questionnaire.

A self-reported before-after study where combined with objective quality indicator measurement by the instructors. No examination was conducted prior to enrolment to the study. All participants that gave informed consent anonymously answered a written survey prior to both live drills (additional file 2). The study design is depicted in figure 4. The two questionnaires were linked without compromising anonymity and self-efficacy and reaction to the training was calculated. Each question relating to self-efficacy was scored on a 7-point Likert scale with points labelled "Did not work" (1) through "Worked excellent" (7). During both exercises, one instructor documented quality indicators such as over- and undertriage rates. Triage accuracy was calculated according to allocated priority at casualty clearing station (first simulation; without TAS-triage) and according to TAS-triage tags (last simulation; with TAS-triage). The instructors also measured quality indicator: time from "scene secured" to all patients triaged (minutes).

The Regional Committee for Medical and Health Research Ethics deemed that approval was unnecessary (2009/1390a). The Norwegian Social Science Data Services approved the study (22991/2/MAB). STROBE guidelines for reporting observational studies and the SQUIRE publication guidelines for quality improvement in health care were utilized in the drafting of this report [9,10].

### Data analysis

Data were collected in Excel spreadsheets (^© ^2007 Microsoft Corporation) and analysed in STATA/SE 10.1 (^© ^Statacorp, TX, USA). Overtriage was fraction of patients given too high priority, whereas undertriage was fraction of patients given too low priority. Continuous variables measured before and after the TAS course were compared using the paired-sample t-test.

## Results

### Descriptive

A total of 110 emergency service professionals attended one of the four courses and 93 learners (85%) answered the questionnaires. Among the study-participants, 26 (28%) worked in healthcare (nurse, ambulance, other), 47 (51%) were fire fighters, 13 (14%) learners were police officers and 7 (7%) had "other" backgrounds.

The mean participant age was 39 years (range 20-62), 84% were men and the median working experience was 8 years (range 0-34).

### Triage accuracy and time expenditure

48% of the learners confirmed that a system for major incident triage existed in their service, whereas 27% had access to triage tagging equipment. Triage accuracy with and without the use of TAS-triage is depicted in table 1. Time from "scene secured" to all patients were triaged was mean 22 minutes (range 15-32) before and mean10 minutes (range 5-21) in the simulation after the course was attended.

**Table 1:** Over- and undertriage without and with the use of TAS-Triage

	**Without TAS-triage**		**With TAS-triage**	
	
	**Overtriage**	**Undertriage**	**Overtriage**	**Undertriage**
	
**Course**				
	
1*	3/20 (15%)	1/20 (5%)	0/20 (0%)	0/20 (0%)
	
2	3/20 (15%)	3/20 (15%)	0/20 (0%)	0/20 (0%)
	
3	2/17 (11,8%)	2/17 (11,8%)	0/17 (0%)	0/17 (0%)
	
4	1/17 (5,9%)	3/17 (17,6%)	0/21 (0%)	0/21 (0%)
	
Total	9/74 (12,2%)	9/74 (12,2%)	0/78 (0%)	0/78 (0%)

Note:

Triage accuracy = mistriage/total patients (n)

*) Simulation was conducted without paediatric mannequins/patients, but with access to Paediatric Triage Tape Stretcher

### Self-efficacy and reaction to training

The slap-wrap triage tags were reported to work well, median = 6 (IQR 6-7). The learners found the paediatric triage tape stretcher feasible, median = 5 (IQR 4-6). Self-efficacy before and after the TAS-course is depicted in table 2.

**Table 2:** Self-efficacy before and after the TAS-course

Question			Likert scale: "Did not work" (1) through "Worked excellent" (7)
		(n)	mean (95% CI)
"How did triage work?"	Before course	87	4,9 (4,6-5,1)
	After course		5,9 (5,7-6,1)*

"How did interdisciplinary cooperation of triage work?"	Before course	88	4,9 (4,7-5,2)
	After course		5,8 (5,6-6,0)*

"How did triage tagging work?"	Before course	83	3,8 (3,4-4,3)
	After course		6,0 (5,8-6,1)*

Note:

CI = Confidence Interval; *) p < 0,001

Each question was scored on a 7-point Likert scale with points labelled "Did not work" (1) through "Worked excellent" (7)

## Discussion

Emergency service personnel reported a significantly increased self-efficacy in major incident triage after being taught the TAS-concept (Table 2). Our modified triage Sieve and PTT were time efficient and accurate (Table 1) in allocating patient priority in simulated major incidents. We found the TAS-concept for major incident triage to be feasible for Norwegian emergency service personnel.

Optimal major incident management rely on qualified rescue workers. An analysis of the medical response to the 2005 London terrorist bombings found that triage accuracy improved when the triage sieve was performed by trained, experienced personnel working in their usual environment [2]. The TAS-concept emphasize inter-disciplinary cooperation and all emergency service professionals (healthcare, police and fire fighters) are taught triage techniques. In a study of British police officers attending a tactical medicine course, Kilner et al. found that learners were able to make accurate triage decisions after being provided triage Sieve decision-making material [11].

Major incident triage remains a neglected field for scientific inquiry [6], and determining effectiveness of triage tools has been identified as a critical area for research [12]. Further, the demonstration of proficiency in the use of triage systems, have been regarded as a core clinical competency for health care personnel [13]. The optimal triage algorithm is characterized by simplicity, time efficiency, predictive validity, reliability and accuracy to minimize mistriage [14]. In a review of published experience with terrorist bombings, Frykberg and Tepas found a mean overtriage rate of 59%. They also identified a linear relationship between overtriage rate and critical mortality, indicating that inappropriate consumption of constrained resources impairs the management of the severely injured [15]. In a prospective validation, Wallis et al. found the PTT to yield acceptable over- and undertriage rates [16].

There are several limitations to this study. During the four full-scaled simulations, we achieved an unrealistically high triage accuracy using the TAS-triage concept. In a chaotic environment, accurate measurement of vital data such as respiratory and heart rate can be unfeasible. Vital data are denoted per minute and often there will be no time to do a full assessment. Our patient information cards provided the learners with an unrealistically easy access to accurate vital data and thus biased the triage accuracy calculations. Further, the paediatric triage was biased as all children were simulated with static mannequins formally in need for a stretcher (minimum (P2) urgent (yellow)). Optimally, our concept for major incident triage should not have been evaluated in simulations, as they can only serve as approximates of complex real incidents. However, research on disaster medicine is largely descriptive as major incidents are virtually impossible to study via randomized controlled trials. Further, our study utilised self-reported variables as measures of effect, although they vary in accuracy [17]. In order to address this limitation, the instructors provided externally rating of quality indicators such as triage accuracy and time expenditure to increase objectivity. Until real-incident experience with the TAS-concept is objectively measured; we need to ensure that our models are feasible, time efficient and accurate in full-scale simulations.

We adapted and modified the MIMMS triage concept in order to increase feasibility for Norwegian emergency services. MIMMS is successfully taught outside the UK and modules are modified to established principles for disaster management [18].

We decided to omit capillary refill from our modified triage Sieve as decreased temperature and dark conditions significantly impairs the field assessment of capillary refill time [19,20]. As a second modification, we renamed the "dead category" to "lifeless" as jurisdictional restrictions apply to defining death in Norway.

Third, we replaced the MIMMS paper tags with slap-wrap reflective triage tags. Paper tags have well-known limitations such as problematic identification in sub-optimal lightning and the tags are likely to perish in our sub-arctic climate [21,22]. Further, paper tags deviate from familiar routines when stress suggests simple and field-friendly solutions. The time used filling in tags soaked in blood may be better utilized caring for severely injured patients. If the situation allows documentation, existing ambulance reports should be utilized to ensure familiarity and avoidance of tags containing information of little value [23]. In our study only 27% had access to triage tagging equipment and only 48% confirmed the existence of a system for major incident triage.

Glow sticks have been documented to contribute to rapid and accurate casualty collection in suboptimal lighted simulations although their shelf-life is unknown [21].

Reflective slap wrap triage tags (figure 3) are a low-cost alternative that tolerate wet and windy conditions and where shelf-time is almost unlimited. Further, they represent a secure tag attachment avoiding confusion from lost tags.

Several training programmes in disaster management exists, but the majority are time consuming and focus on the medical aspect of major incident management [24].

When major incidents occur, a variety of local or national agencies providing various necessary services work together to improve outcome. It seems evident that effective major incident management relies on clear and effective inter-disciplinary communication, especially of critical information such as triage priority of patients. Major incident triage is dynamic and patients are repeatedly re-triaged along the evacuation chain and through the receiving hospital until definitive treatment is received. In Norway, a train accident near Aasta killed 19 people whereas 67 passengers survived. Approximately 600 personnel from different 11 services participated in the initial management of this major incident [25]. A review of the World Trade Center attack in 2001 concluded that "the lack of communication resulted in more problems than all other factors combined" [26]. Further, during a major aircraft incident in UK, the simultaneous use of several different triage-labelling systems contributed to confusion [27]. A triage concept with uniform instructions and standardized triage tagging would alleviate on-scene confusion and national standards has been called for both in the US and Australia [14,28]. In Norway, the lack of a standard major incident triage concept that is nationally accepted, reliable and validated remains a gap in our major incident preparedness.

## Conclusions

Major incident triage skills can be effectively taught to multi-disciplinary emergency service professionals using a combination of lectures and practical simulations in a two-day course. Our modified triage Sieve tool provides acceptable accuracy in allocating priority during simulated major incidents and may serve as a candidate for a future national standard for major incident triage.

## Competing interests

Declared. The TAS-courses are funded and organized by the Norwegian Air Ambulance Foundation. Trond Vigerust is a hired consultant for LESS, a manufacturer of emergency stretchers. All other authors declare no conflict of interest.

## Authors' contributions

MR, HML, AJK, TV and JEA conceived the study. MR, AJK, TV and JEA designed the study. JEA supervised the data collection. TV, AJK and MR managed the collected data. MR performed the analysis and drafted the manuscript. All authors interpreted data and critically revised the manuscript. All authors have read and approved the final manuscript.

## Pre-publication history

The pre-publication history for this paper can be accessed here:

http://www.biomedcentral.com/1471-227X/10/17/prepub

## Supplementary Material

Additional file 1 **Example of patient information card**. Status inside bus wreck and at casualty clearing station. Click here for file

Additional file 2 **Questionnaire**. Word file containing questionnaire (in Norwegian language). Click here for file
