# Supplementary material for: A concept for major incident triage: full-scaled simulation feasibility study
Source: BMC Emerg Med. 2010 Aug 11;10:17. doi: 10.1186/1471-227X-10-17 (PMC2928192; doi:10.1186/1471-227X-10-17)
Supplement: Additional file 1 — Example of patient information card. Status inside bus wreck and at casualty clearing station. [file 1471-227X-10-17-S1.PPT]

## Slide 1
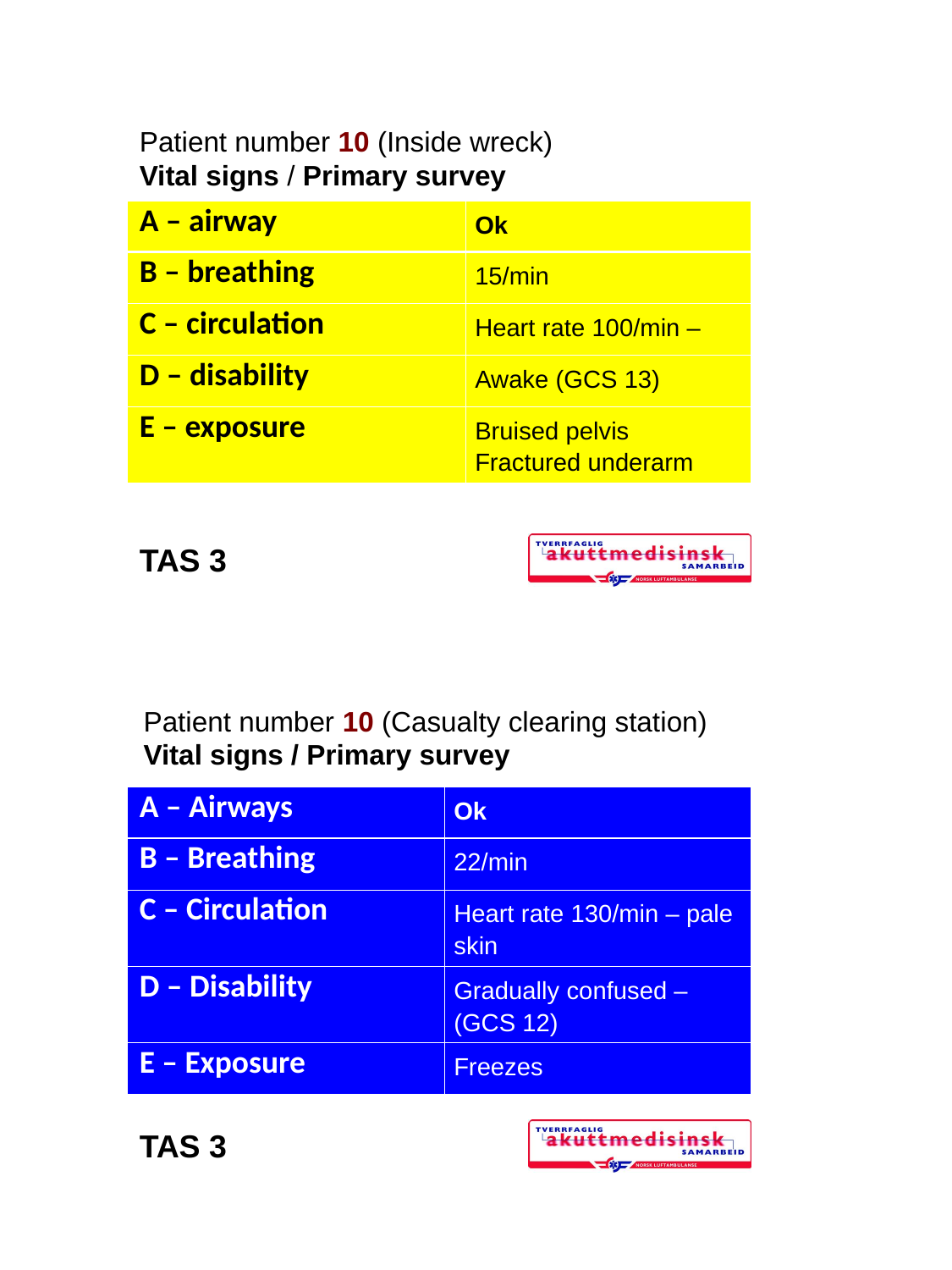

Patient number 10 (Inside wreck)
Vital signs / Primary survey
| A – airway | Ok |
| --- | --- |
| B – breathing | 15/min |
| C – circulation | Heart rate 100/min – |
| D – disability | Awake (GCS 13) |
| E – exposure | Bruised pelvis Fractured underarm |
TAS 3
Patient number 10 (Casualty clearing station)
Vital signs / Primary survey
| A – Airways | Ok |
| --- | --- |
| B – Breathing | 22/min |
| C – Circulation | Heart rate 130/min – pale skin |
| D – Disability | Gradually confused –(GCS 12) |
| E – Exposure | Freezes |
TAS 3
