# Supplementary material for: A concept for major incident triage: full-scaled simulation feasibility study
Source: BMC Emerg Med. 2010 Aug 11;10:17. doi: 10.1186/1471-227X-10-17 (PMC2928192; doi:10.1186/1471-227X-10-17)
Supplement: Additional file 2 — Questionnaire. Word file containing questionnaire (in Norwegian language). [file 1471-227X-10-17-S2.DOC]

**
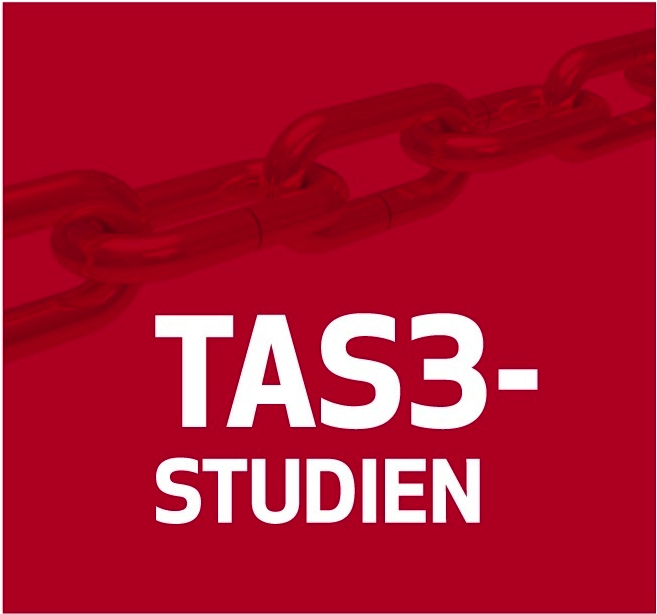
**

Dette er et spørsmål til deg som TAS3 kursdeltaker om å delta i en **spørreskjemaundersøkelse** som søker å kartlegge kvaliteten på deler av **TAS3 kurset.**

Vi gjennomfører denne studien for å påvise eventuelle områder med forbedringspotensial samt å vurdere effekten av nyinnførte tiltak.

Studien ledes av forsker Marius Rehn og resultatene vil bli publisert i et vitenskapelig tidsskrift. Stiftelsen Norsk Luftambulanse er ansvarlige for studien. Det vil ta ca. 5 minutter å besvare en spørreundersøkelse **etter øvelse 1** (starten av kurset) og **etter øvelse 2** (slutten av kurset). I tillegg vil TAS3 instruktørene dokumentere resultatene av øvelsene.

**Hva skjer med informasjonen om deg?** Spørreskjemaet vil ikke inneholde ditt navn, men vi spør om en del indirekte personidentifiserende opplysninger som kjønn, alder og yrke. I publiseringen av undersøkelsen vil alle informanter være anonyme. Anonymiseringen av spørreskjemaene vil skje ved prosjektslutt 31.08.2010.

**Frivillig deltakelse:** Det er frivillig å delta i denne undersøkelsen. Du er velkommen til å delta på TAS3 kurset uavhengig av om du deltar i spørreundersøkelsen eller ikke. Det regnes som et samtykke til å delta på undersøkelsen dersom du fyller ut vedlagte spørreskjemaer. Ved spørsmål kan du kontakte Marius Rehn; mob: 90784044, e.post: [marius.rehn@snla.no](mailto:marius.rehn@snla.no)

På forhånd takk, hilsen oss i TAS3

**TAS3–Studien; Spørsmål etter FØRSTE øvelse**

| 1) Hva er din **alder**? | Antall hele **år**: **_____________** |
| --- | --- |
| 2) Antall **år** du har jobbet i utrykningsyrket? | Antall hele **år**: **_____________** |
| Sett ett–1–kryss i det alternativet **DU** mener passer best | |
| 3) Hvilket **kjønn** har du? | **Mann**:  **Kvinne**: |
| 4) Hva er din **yrkeskategori**? | **Helse**: Lege  Sykepleier  Paramedic  Ambulansearbeider  Annen helsearbeider  **Brann**:  **Politi**:  **Bilberger**:  **Annet**: |
| 5) Eksisterer det i din tjeneste, et system for **sortering og prioritering (triage)** av pasienter involvert i en stor ulykke? | **Ja**:  **Nei**:  **Vet ikke**: |
| 6) Er **merkeutstyr for pasientsortering** (triage) tilgjengelig i din tjeneste? | **Ja**:  **Nei**:  **Vet ikke**: |
| 7) Hvis ja;  Hva slags **type merkeutstyr** finnes? | **Predoc**  **Lysstaver**  **Refleksbånd**  **Annet**  **TAS–Triage bånd** |
| 8) Finnes det et **bårelager** i ditt område? | **Ja**:  **Nei**:  **Vet ikke**: |
| 9) Hvor mange **bårer** er tilgjengelig? | **Under 5**  **5–15**  **16–25**  **Over 25**  **Vet ikke** |
| 10) Hvor lang tid (i snitt) tror DU det tar og få disse **bårene frem til skadested**? | **Under 15 min**  **15–30 min**  **31–45 min**  **Over 45 min**  **Vet ikke** |
| 11) Hvilket **tiltakskort/setning** benytter **DU**? | **PBS**  **OBBO**  **OSATT**  **Annet**  **Benytter ikke** |
| Resten av spørsmålene er relatert til erfaringer **DU** har gjort i forbindelse med den **første TAS–øvelsen** | |
| **Pasientprioritering** | |
| 12) Hvordan opplevde du at **sortering og prioritering (”triage”)** av pasientene fungerte? | **1** **2** **3** **4** **5** **6** **7**  **(**1**=*Fungerte ikke*,** 7**=*Fungerte utmerket*)** |
| 13) Hvordan opplevde du at den **tverrfaglige samhandlingen av triage** fungerte? | **1** **2** **3** **4** **5** **6** **7**  **(**1**=*Fungerte ikke*,** 7**=*Fungerte utmerket*)** |
| 14) Hvordan opplevde du at **merking av pasientene** fungerte? | **1** **2** **3** **4** **5** **6** **7**  **(**1**=*Fungerte ikke*,** 7**=*Fungerte utmerket*)** |
| 15) Hvordan opplevde du **tidsforbruket til triage**? | **1** **2** **3** **4** **5** **6** **7**  **(**1**=*Ikke effektivt*,** 7**=*Svært effektivt*)** |
| **Pasientevakuering** | |
| 16) Hvordan opplevde du at **pasientevakuering/pasienttransport** fungerte? | **1** **2** **3** **4** **5** **6** **7**  **(**1**=*Fungerte ikke*,** 7**=*Fungerte utmerket*)** |
| 17) Hvordan opplevde du at den **tverrfaglige samhandlingen av pasientevakuering/pasienttransport** fungerte? | **1** **2** **3** **4** **5** **6** **7**  **(**1**=*Fungerte ikke*,** 7**=*Fungerte utmerket*)** |
| 18) Hvordan opplevde du at **transportmateriellet** fungerte? | **1** **2** **3** **4** **5** **6** **7**  **(**1**=*Fungerte ikke*,** 7**=*Fungerte utmerket*)** |
| 19) Hvordan opplevde du **tidsforbruket til pasientevakuering/pasienttransport**? | **1** **2** **3** **4** **5** **6** **7**  **(**1**=*Ikke effektivt*,** 7**=*Svært effektivt*)** |
| 20) Hvordan opplevde du at pasienten ble **skjermet mot generell nedkjøling (hypotermi)**? | **1** **2** **3** **4** **5** **6** **7**  **(**1**=*Ikke skjermet*,** 7**=*Utmerket skjermet*)** |

**Nå er du ferdig med del 1!**

**Takk for at du svarte på det første spørreskjemaet!**

**TAS3–Studien; Spørsmål etter SISTE øvelse**

| Sett ett–1–kryss i det alternativet **DU** mener passer best | |
| --- | --- |
| **TAS–Triage** | |
| 21) Hvordan opplevde du at **sortering og prioritering (”triage”)** av pasientene fungerte? | **1** **2** **3** **4** **5** **6** **7**  **(**1**=*Fungerte ikke*,** 7**=*Fungerte utmerket*)** |
| 22) Hvordan opplevde du at den **tverrfaglige samhandlingen av triage** fungerte? | **1** **2** **3** **4** **5** **6** **7**  **(**1**=*Fungerte ikke*,** 7**=*Fungerte utmerket*)** |
| 23) Hvordan opplevde du at **merking av pasientene** fungerte? | **1** **2** **3** **4** **5** **6** **7**  **(**1**=*Fungerte ikke*,** 7**=*Fungerte utmerket*)** |
| 24) Hvordan opplevde du **tidsforbruket til triage**? | **1** **2** **3** **4** **5** **6** **7**  **(**1**=*Ikke effektivt*,** 7**=*Svært effektivt*)** |
| 25) Hvordan opplevde du at **TAS–Triage flytskjema** fungerte? | **1** **2** **3** **4** **5** **6** **7**  **(**1**=*Fungerte ikke*,** 7**=*Fungerte utmerket*)** |
| 26) Hvordan opplevde du at **TAS–Triage merkebånd** fungerte? | **1** **2** **3** **4** **5** **6** **7**  **(**1**=*Fungerte ikke*,** 7**=*Fungerte utmerket*)** |
| 27) Hvordan opplevde du at **TAS–Triagetape (barnetriage)** fungerte? | **1** **2** **3** **4** **5** **6** **7**  **(**1**=*Fungerte ikke*,** 7**=*Fungerte utmerket*)** |
| **TAS–OPEN (Pasientevakuering)** | |
| 28) Hvordan opplevde du at **pasientevakuering/pasienttransport** fungerte? | **1** **2** **3** **4** **5** **6** **7**  **(**1**=*Fungerte ikke*,** 7**=*Fungerte utmerket*)** |
| 29) Hvordan opplevde du at den **tverrfaglige samhandlingen av pasientevakuering/pasienttransport** fungerte? | **1** **2** **3** **4** **5** **6** **7**  **(**1**=*Fungerte ikke*,** 7**=*Fungerte utmerket*)** |
| 30) Hvordan opplevde du at **materiellet for pasienttransport** fungerte? | **1** **2** **3** **4** **5** **6** **7**  **(**1**=*Fungerte ikke*,** 7**=*Fungerte utmerket*)** |
| 31) Hvordan opplevde du **tidsforbruket til pasientevakuering/pasienttransport**? | **1** **2** **3** **4** **5** **6** **7**  **(**1**=*Ikke effektivt*,** 7**=*Svært effektivt*)** |
| 32) Hvordan opplevde du at **TAS–OPEN bårebag** fungerte? | **1** **2** **3** **4** **5** **6** **7**  **(**1**=*Fungerte ikke*,** 7**=*Fungerte utmerket*)** |
| 33) Hvordan opplevde du at **oversikten og systematiseringen på samleplass fungerte**? (med NLA båren og TAS OPEN prinsippene) | **1** **2** **3** **4** **5** **6** **7**  **(**1**=*Fungerte ikke*,** 7**=*Fungerte utmerket*)** |

| **Annet** | |
| --- | --- |
| 34) Hvordan opplevde du at **TAS–Hypotermi bobleplast** fungerte? | **1** **2** **3** **4** **5** **6** **7**  **(**1**=*Fungerte ikke*,** 7**=*Fungerte utmerket*)** |
| 35) Hvordan opplevde du at **TAS–tiltakskort ”Forstått”** fungerte? | **1** **2** **3** **4** **5** **6** **7**  **(**1**=*Fungerte ikke*,** 7**=*Fungerte utmerket*)** |
| 36) I arbeidet for at utrykningsetatene skal vise samhandling og være effektive; hvor viktig mener du at et **felles språk for utrykningsetatene** er? | **1** **2** **3** **4** **5** **6** **7**  **(**1**=*Ikke viktig,*** 7**=*Svært viktig*)** |

**Takk for at du tok deg tid til å besvare spørsmålene**

**Dine erfaringer er viktige…**

**Vennlig hilsen oss i TAS3**
